# *In situ* terahertz monitoring of an ice ball formation during tissue cryosurgery: a feasibility test

**DOI:** 10.1117/1.JBO.26.4.043003

**Published:** 2021-01-27

**Authors:** Arsen K. Zotov, Arsenii A. Gavdush, Gleb M. Katyba, Larisa P. Safonova, Nikita V. Chernomyrdin, Irina N. Dolganova

**Affiliations:** a Institute of Solid State Physics of the Russian Academy of Sciences, Chernogolovka, Russia; b Prokhorov General Physics Institute of the Russian Academy of Sciences, Moscow, Russia; c Bauman Moscow State Technical University, Moscow, Russia; d Sechenov First Moscow State Medical University (Sechenov University), Institute for Regenerative Medicine, Moscow, Russia

**Keywords:** cryoablation, cryotherapy, terahertz biophotonics, tetrahertz pulsed spectroscopy, ice ball formation

## Abstract

**Significance:** Uncontrolled cryoablation of tissues is a strong reason limiting the wide application of cryosurgery and cryotherapy due to the certain risks of unpredicted damaging of healthy tissues. The existing guiding techniques are unable to be applied *in situ* or provide insufficient spatial resolution. Terahertz (THz) pulsed spectroscopy (TPS) based on sensitivity of THz time-domain signal to changes of tissue properties caused by freezing could form the basis of an instrument for observation of the ice ball formation.

**Aim:** The ability of TPS for *in situ* monitoring of tissue freezing depth is studied experimentally.

**Approach:** A THz pulsed spectrometer operated in reflection mode and equipped with a cooled sample holder and *ex vivo* samples of bovine visceral adipose tissue is applied. Signal spectrograms are used to analyze the changes of THz time-domain signals caused by the interface between frozen and unfrozen tissue parts.

**Results:** Experimental observation of TPS signals reflected from freezing tissue demonstrates the feasibility of TPS to detect ice ball formation up to 657-μm depth.

**Conclusions:** TPS could become the promising instrument for *in situ* control of cryoablation, enabling observation of the freezing front propagation, which could find applications in various fields of oncology, regenerative medicine, and THz biophotonics.

## 1 Introduction

Terahertz (THz) pulsed spectroscopy (TPS) is known as a promising tool of biophotonics and medical diagnosis.^1^^–^^4^ It demonstrates strong potential of study and differentiate various conditions of tissues, both *ex vivo* and *in vivo*,^5^^–^^9^ and imaging and characterization of neoplasms in different localizations,^10^^–^^14^ by means of endogenous markers of pathology, such as water content. However, high concentration of free and bound water in tissues restricts the penetration depth of THz waves by only several hundreds of microns.^1^ To overcome this limitation, different approaches can be applied. Among them are such techniques as tissue dehydration,^15^ paraffin-embedding,^16^ lyophilization,^17^ compression,^18^ immersion optical clearing,^19^^,^^20^ and freezing.^21^^–^^24^ It was demonstrated that the latter one helps to increase the THz-wave penetration up to 1 mm, changing at the same time dielectric properties of tissues in THz range.^23^ Along with reduction of absorption, low temperatures alter the refractive index within certain limits, thus, yielding the dielectric contrast between tissues in different states. This opens further horizons of TPS applications, in particular, for monitoring of freezing depth in tissues during their cryosurgery.

Uncontrolled cryoablation of living tissues is a severe problem of modern cryosurgery, i.e., application of extreme cold aimed at rapid freezing and thus destroying or renewal of pathological tissues.^25^^–^^29^ Unless cryoablation possesses several advantages, such as relative painlessness, hemostatic effect, short recovering of patients, and immunostimulating effect,^25^^,^^30^^,^^31^ it is associated with certain risks of damaging healthy tissues surrounding the pathology and with possibility of incomplete cell death.^28^ In particular, the use of cryosurgery for the treatment of brain tumors and epilepsy is currently limited by the possible risks for the patient.^32^^–^^34^ Such guiding techniques as ultrasonography,^35^^,^^36^ magnetic resonance, and computer imaging^37^^–^^42^ do not enable the monitoring of the tissue freezing *in situ* with high spatial resolution near the cryoprobe. Alternative approach is based on measuring of tissue temperature using thermocouples (TCs) combined with cryoprobe.^43^ However, it allows controlling of the tissue condition only at the cryoprobe–tissue interface. Therefore, cryosurgery still needs guiding approaches for the efficient control of ice ball formation.

In this work, we study the ability of TPS to solve this problem. We experimentally demonstrate the changes of THz time-domain signal during tissue freezing, using *ex vivo* bovine adipose specimen and laboratory spectrometer equipped with special sample holder. Processing of THz pulsed signal within 45 s demonstrates the ability to detect the movement of freezing front up to the depth of 657  μm. The results of this work reveal the potential of TPS to control the initial stage of ice ball formation during tissue cryoablation.

## 2 Experimental Setup

**Fig. 1:**
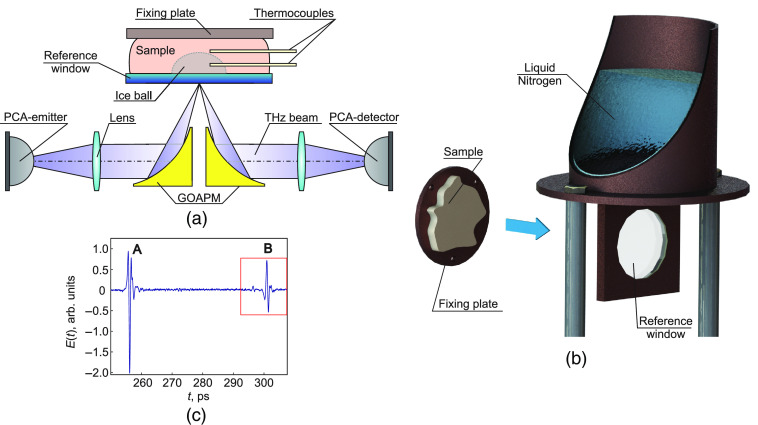
Schematic of the *ex vivo* TPS measurement of the tissue freezing: (a) a reflection-mode measurement unit of the THz pulsed spectrometer, PCA stands for a photoconductive antenna, GOAPM stands for a gold off-axis parabolic mirror; (b) a sample holder; (c) an example of the sample time-domain signal E(t) of the THz pulsed spectrometer, where A and B stand for pulses reflected from the interfaces free space–reference window and reference window–sample, respectively.

To experimentally test the feasibility of TPS to detect the freezing depth in biological tissues, we use a laboratory THz pulsed spectrometer, which was described in detail in Ref. 44. It operates in a reflection mode and has a maximum spectral operating range from about 0.05 to 4.0 THz and a maximum spectral resolution of about 0.002 THz. The THz wave features s-polarization. To avoid the impact of water vapors on the measurements, the spectrometer is purged with nitrogen gas. It is also equipped with a sample holder (Fig. 1). The latter consists of a reference window made of sapphire, which on the one hand is placed on the THz beam path in contact with the tissue sample [see Fig. 1(a)]. On the other hand, it is inserted in the metal frame connected with the reservoir filled with liquid nitrogen prior to the measurements [Fig. 1(b)]. Thus, due to high thermal conductivity of metal and sapphire, the window possesses low and stable temperature during the measurements.

As a tissue sample, we use *ex vivo* bovine visceral adipose tissue because of its relatively homogeneous structure. The sample thickness is near 30 mm enabling prolonged duration of the freezing process. By two off-axis parabolic mirrors, the incident THz beam is focused on the interface between the reference window and the tissue; the reflected beam is collimated to the detector. Generation and detection of THz pulses are provided by two photoconductive antennas (PCAs). A typical form of the reflected THz time-domain signal is shown in Fig. 1(c). The sequence of pulses includes signal (A) reflected from free space–window interface and signal (B) reflected from the window–sample interface. Since we are interested in the latter one, the scanning range of the spectrometer is adjusted at this pulse (marked with red rectangle).

The measurements are performed at room temperature 24°C. However, the sample is preliminary heated to the temperature 37°C aimed at the slowing down the initial stage of freezing. After the contact between the tissue and the cooled window, THz pulsed signal is constantly recorded with the period of 15 s until the sample is completely frozen. For additional control of sample freezing, its temperature is measured by the inserted TCs.

## 3 THz Pulsed Spectroscopy of a Freezing Tissue

### 3.1 THz Dielectric Properties of a Sample

The sample refractive index was characterized before and after freezing. For this purpose, the complex amplitude of the incident THz field is considered to have the form $E(t)=A_{0}\exp(i\phi )$, where $A_{0}$ and ϕ are the amplitude and phase of the THz field, respectively. Assuming the negligible losses in the reference window and the normal incidence of THz beam, the signal amplitudes $A_{1}$ and $A_{2}$ of pulses reflected from the first and second interfaces [see wavelets (A) and (B) in Fig. 1(c)] are described by the Fresnel coefficients

$$A_{1}=R_{01}A_{0},$$ (1)

$$A_{2}=T_{01}R_{12}T_{10}A_{0},$$ (2)

where $R_{mk}$ and $T_{mk}$ are the Fresnel coefficients of amplitude reflection and transmission between m’th and k’th media, which for s-polarized wave are determined by the corresponding refractive indices $n_{m}$ and $n_{k}$

$$R_{mk}=\frac{n_{m}-n_{k}}{n_{m}+n_{k}},$$ (3)

$$T_{mk}=\frac{2n_{k}}{n_{m}+n_{k}}.$$ (4)

In this work, refractive indices for free space and reference window are $n_{0}=1$ and $n_{1}=3.07$, respectively, at 1-THz frequency.^45^ Estimating the relation $\left|A_{2}/A_{1}\right|$ from the measured TPS waveforms and accounting the dispersion relation, the values of the sample refractive index $n_{2}$ at 1-THz frequency were determined for two conditions of the tissue, i.e., $n_{R}=1.84\pm 0.01$ at room temperature and $n_{F}=1.37\pm 0.01$ after freezing. Thus, freezing of the adipose tissue leads to significant changes of its refractive index. The value $n_{F}$ is used further for estimation of the freezing depth z according to the relation $z=tc/(2n_{F})$, where t is the temporal position of the detected freezing depth, c is speed of light in free space.

### 3.2 Analysis of THz Signal Spectrograms

According to the measurement of sample temperature, the freezing depth of 4 mm was reached in <1  min. This depth significantly exceeds the typical values of THz penetration depth in frozen biological tissues.^23^ Thus, only several THz waveforms can be recorded during this stage of the analyzed process. To increase the number of detected signals, the accumulation time and discretization of the spectrometer should be reduced.

**Fig. 2:**
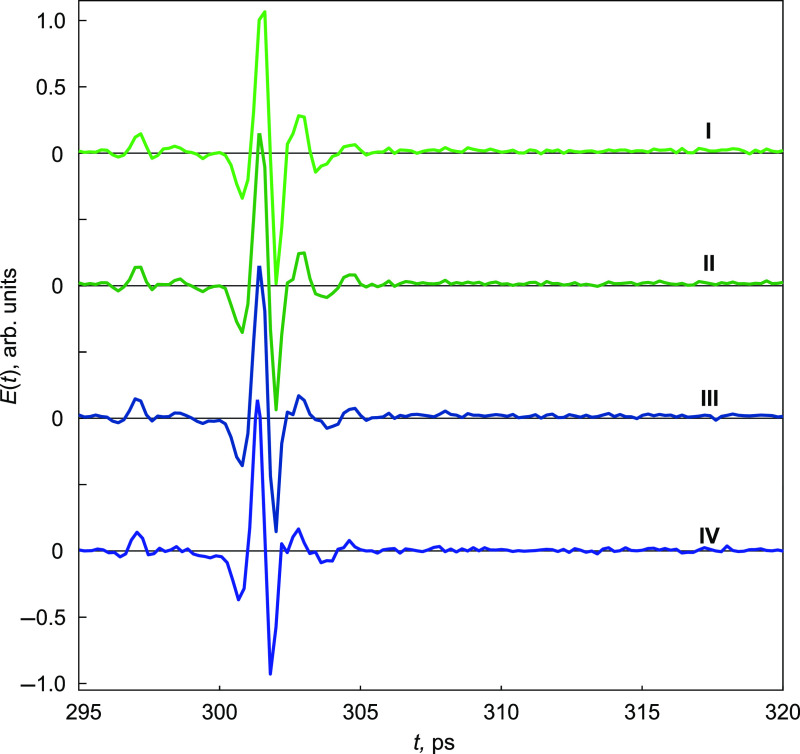
The waterfall of THz time-domain signals reflected from the window–tissue interface and detected by the spectrometer during the tissue freezing. Signals I, II, III, and IV correspond to t=0, 15, 30, and 45 s moments of freezing, respectively.

Figure 2 shows the sequence of THz waveforms detected at (I) 0 s, (II) 15 s, (III) 30 s, and (IV) 45 s from the beginning of the sample cooling. At the initial moment t=0 of the ice front propagation, the temporal width of first THz pulse, caused by the window–tissue interface, is about 5 ps, which corresponds to ∼0.5  mm of the sample depth. Within this tissue layer, the second pulse caused by the existence of the ice front interface, is probably overlapped by the first one. Its amplitude is expected to be significantly smaller, since the dielectric contrast between frozen and unfrozen tissue is lower than that for the tissue and the reference window. Thus, it is difficult to estimate directly from the THz waveforms the moving position of the second pulse.

**Fig. 3:**
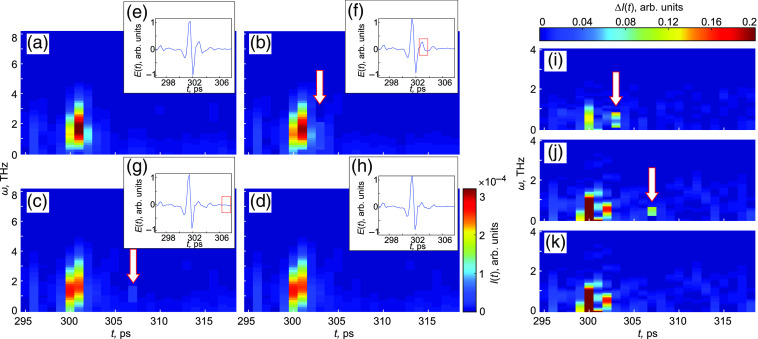
Analysis of THz time-domain signals detected during the tissue freezing. (a)–(d) Signal intensity spectrograms, where white arrows indicate the positions of second pulses reflected from the freezing front; (e) and (f) the temporal amplitudes of the signals, where red rectangles mark the same positions; (a) and (e) correspond to the moment t=0; (b) and (f) 15 s; (c) and (g) 30 s; and (d) and (h) 45 s; (i)–(k) the differences ΔI(t) between the normalized spectrogram $I_{n}(t)=I(t)/\max[I(t)]$ at t=15, 30, and 45 s and the initial normalized spectrogram at t=0.

To overcome this problem, we analyze the signal intensity spectrograms in addition to their temporal waveforms. Figures 3(a)–3(d) show the spectrograms of the measured signals shown in the insets [Figs. 3(e)–3(h)]. Comparing them between each other, it is possible to detect the temporal position of the pulse reflected from the ice front. Figures 3(i)–3(k) show the difference ΔI(t) between the normalized spectrogram $I_{n}(t)=I(t)/\max[I(t)]$ at t=15, 30, and 45 s and the initial normalized spectrogram at t=0. Obviously, the first pulse is altering during the first 30 s, then its appearance remains stable. We can clearly observe the presence of the second pulse at t=15 and 30 s, which indicates the freezing depths of z=328  μm and z=657  μm, respectively; then, its amplitude becomes lower due to absorbtion, which does not allow us to detect it. Therefore, during 30 s starting from the tissue cooling, TPS provides an ability to analyze the ice ball formation and estimate the freezing depth. It should be mentioned that time-domain THz signals feature rather weak changes at the determined moments, not allowing us to evaluate them directly. To demonstrate this, the detected positions of the second pulses at t=15 and 30 s are marked with red rectangles on the THz waveforms [see Figs. 3(f) and 3(g)]. Due to the absence of second pulses at t=0 and 45 s, there are no marks on Figs. 3(e) and 3(h).

## 4 Results and Discussion

**Fig. 4:**
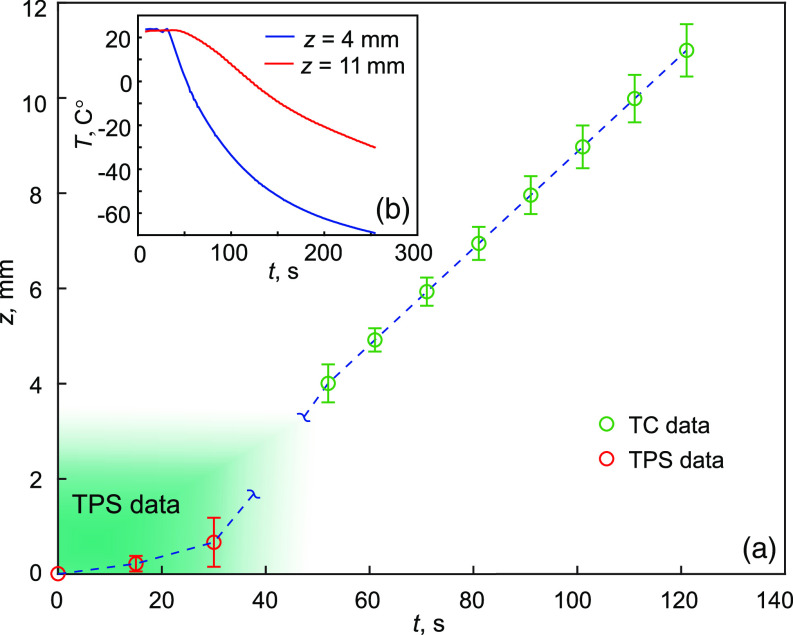
An estimation of the tissue freezing depth. (a) The combination of TPS measurements (points marked with red color) and data obtained by TCs (points marked with green color), here, TC stands for thermocouple; (b) temperature changes of the freezing tissue at depths of 4 and 11 mm from the reference window. Error bars represent ±2σ confidential interval of measurements.

The estimation of the tissue freezing depth is shown in Fig. 4, where the values obtained by TPS are combined with the direct measurements of the sample temperature. Figure 4(b) demonstrates the temperature change at two depths 4 and 11 mm from the window–tissue interface. The slowdown of the freezing process at the beginning can be explained by the initial cooling of the tissue from 37°C to 0°C. In our feasibility study, the maximal freezing depth observed by TPS is limited by the value 657  μm. However, we believe that it could be increased using THz time-domain spectrometer with more efficient performance, particularly, scanning rate, which would yield the reduction of the detection period and increasing of the temporal resolution.

Though the penetration depth of THz waves even in frozen tissue remains extremely low, the detection of the freezing depth by TPS could provide rather high precision of monitoring ice front propagation, enabling the observation and control of cryoablation and cooling of cell and artificial tissue structures, and agglomeration of cell spheroids. Thus, it could become a beneficial research instrument in regenerative medicine and microbiology.

THz technologies are still far from clinical application, suffering from a list of problems, such as low performance of THz sources and detectors, and low spatial resolution due to high absorbtion and scattering.^46^^,^^47^ However, the rapid progress of THz sources and optical components in the recent years ^48^^–^^52^ stimulates the further improvements of THz instruments for biophotonics. A strong limitation is caused by the absence of efficient waveguides and fibers.^53^ Nevertheless, the sapphire shaped crystal waveguides, exploiting both anti-resonant reflecting optical and photonic crystal waveguidance principles, have certain perspectives for delivering THz pulses with relatively low losses and dispersion;^44^^,^^54^ thus, they could form the basis for further developments of THz instruments for biophotonics and medicine. In addition, sapphire features high thermal conductivity at cryogenic temperatures, being more efficient material for cryosurgery than metal (copper, titanium nickelide, and stainless steel), since it enables higher tissue freezing rate.^53^ Combination of these advantages opens future perspectives for the appearance of sapphire probes for controlled cryoablation.

Cryosurgery demonstrates high potential in oncology, in particular, to remove neoplasms and metastases of different localizations, such as liver,^37^ breast,^55^ lungs,^40^prostate,^56^ kidney,^57^ stomach,^58^ and skin.^59^ Considering the ability of THz technologies for tumor diagnosis along with currently developing THz instruments and methods for healthy and pathological tissues differentiation,^2^ the combination of THz preoperative characterization of tissue with further TPS-controlled cryoablation could become an effective tool for oncology.

It is worth mentioning that several techniques enable increasing of low penetration depth of THz waves into living tissues. Among them are methods of immersion optical clearing,^19^ which could also be combined with THz freezing depth detection. However, a list of hyperosmotic agents applied for tissue clearing act as cryoprotectors (e.g., glycerol, dimethyl sulfoxide, and propylene glycol), since they substitute the interstitial water. Thus, the particular approaches of their application for increasing of THz-wave penetration into tissues during their freezing, such as concentration of aqueous solutions and exposure time, are the subjects of further studies.

## 5 Conclusion

The performed experimental study demonstrates the feasibility of *in situ* observation of the ice ball formation in tissues using TPS instrumentation. Applying the spectrogram analysis of THz time-domain signals recorded in the reflection-mode of THz pulsed spectrometer during tissue freezing, it is possible to detect the positions of the freezing depth up to 657μm. We believe that the described approach would be helpful for the development of novel approaches and instruments of controlled cryoablation and would be useful for various tasks of oncology, regenerative medicine, microbiology, and further progress of THz biophotonics.

## Code, Data, and Materials Availability

All data generated or analyzed during this study are included in this article.
